# The Protective Efficacy of Total Alkaloids from Nelumbinis Plumula on Irradiation-Induced Oxidative Stress Damage in Human Umbilical Vein Endothelial Cells

**DOI:** 10.3390/antiox15010049

**Published:** 2025-12-30

**Authors:** Junyu Bu, Ziming Xia, Jinrui Zhang, Longhui Yan, Yiming Luo, Zhiyan Zhang, Min Li, Shuchen Liu, Ying Tian

**Affiliations:** 1 College of Chemistry and Materials Science, Hebei University, Baoding 071002, China; bujunyu112@163.com; 2 Beijing Institute of Radiation Medicine, Beijing 100850, China; zmxia22@163.com (Z.X.); zjr13753440232@163.com (J.Z.); yanlonghui111@163.com (L.Y.); 13137200855@163.com (Y.L.); zzyan0911@163.com (Z.Z.); limin82057@163.com (M.L.); liusc118@163.com (S.L.)

**Keywords:** total alkaloid extract of Nelumbinis Plumula, oxidative stress, irradiation, pyroptosis, ROS, caspase-1, GSDMD

## Abstract

A high-purity alkaloid-enriched extract (NPAE) was developed from Nelumbinis Plumula. Beyond quantifying its representative alkaloids and total alkaloid content, this study revealed the novel radioprotective role of NPAE against radiation-induced oxidative stress in human umbilical vein endothelial cells (HUVECs). Pretreatment with NPAE significantly attenuated H_2_O_2_-induced oxidative stress and suppressed irradiation-induced pyroptosis, primarily through restoration of redox homeostasis and inhibition of inflammasome activation. Mechanistic investigations showed that NPAE downregulated the expression of GSDMD-N and cleaved caspase-1, while reducing the secretion of proinflammatory cytokines (IL-18 and IL-1β). These results demonstrate that NPAE effectively alleviates oxidative damage and prevents pyroptosis in endothelial cells, highlighting its potential as a promising phytotherapeutic agent for protection against ionizing radiation injury.

## 1. Introduction

The advancement of nuclear energy and technology has significantly contributed to the growth in society and the economy. However, it also generates ionizing radiation (IR), which poses potential risks. As an obvious physical carcinogen, its biological effects are strongly influenced by radiation type, radiation dose, individual susceptibility, and other factors [1]. When improperly applied, it can induce acute radiation damage in sensitive target organs such as the bone marrow and intestines, while also triggering systemic pathological alterations [2,3]. These effects may result in hematologic dysfunction [4] and other physiological disturbances [5].

IR exerts its detrimental effects through both direct and indirect mechanisms, leading to biomolecule damage such as lipid peroxidation, protein oxidation, and DNA damage (strand breaks) [6]. These molecular lesions can subsequently trigger cellular malfunction and activate diverse cell death pathways, including apoptosis, pyroptosis, ferroptosis, and others [7,8]. Given that water constitutes the predominant component of biological systems, most intracellular biomolecules are situated in an aqueous microenvironment [9]. Consequently, the radiolysis of water induced by IR indirectly generates substantial reactive species, including peroxyl radicals (•OH), hydrogen peroxide (H_2_O_2_), and superoxide anion (O_2_^−^), which constitute major reactive oxygen species (ROS) [10]. Emerging evidence indicates that ROS serve as crucial signaling molecules regulating diverse physiological processes with significant implications for human health and immune homeostasis [11,12]. Notably, the function of ROS as pivotal upstream regulators of NLRP3 inflammasome activation [13,14]. The underlying molecular mechanism involves a redox-sensitive cascade: elevated intracellular ROS oxidize reduced thioredoxin (Trx), leading to dissociation of the Trx-TXNIP complex. The liberated thioredoxin-interacting protein (TXNIP) subsequently binds to the NACHT domain of NLRP3, inducing conformational changes that facilitate NLRP3 inflammasome oligomerization and activation [15,16,17]. Therefore, targeting oxidative stress damage and inflammatory reactions represents a paramount therapeutic strategy against IR-induced injury [18].

Advances in our understanding of the molecular mechanisms of radiation-induced damage have facilitated the development of numerous prophylactic and therapeutic interventions. Current strategies target oxidation-sensitive proteins, enhance adaptive responses to oxidative stress, and modulate specific molecular pathways involved in cellular response to IR [19]. Numerous natural products exhibit potent anti-inflammatory and antioxidant properties, which are attributed to their ability to modulate key signaling pathways implicated in radiation-induced damage [20]. Tocopherol analogs exhibited radioprotective properties. Administration of α-tocopherol immediately after radiation exposure significantly enhanced hematopoietic colony-forming units in mouse spleens [21]. Furthermore, delta-tocotrienol demonstrated a protective role against radiation-induced cytotoxicity by suppressing IL-1β-triggered NF-κB/miR-30 signaling, ultimately reducing cell death and improving survival rates in irradiated mice [22]. Oxymatrine significantly activated the MAPK signaling pathway, thereby enhancing hematopoietic stem cell function. This effect not only accelerated recovery from radiation-induced hematological injury but also improved the survival rate of irradiated mice [23]. Ferulic acid demonstrated significant efficacy in mitigating radiation-induced oxidative stress through upregulation of the Nrf2 signaling pathway, while concurrently suppressing hepatic inflammatory responses via inhibition of the JAK/STAT signaling pathway [24]. Therefore, natural products demonstrate considerable potential for development in the field of radioprotection through their multi-target regulatory mechanisms, which encompass the modulation of oxidative stress, intervention in cell death pathways, and regulation of specific signaling molecules, among other biological processes [19,25].

Nelumbinis Plumula (Lianzixin), the embryonic tissue of the mature *Nelumbo nucifera* Gaertn. seed, has been officially recognized in the Chinese Pharmacopeia (Chp) as an important medicinal part of clinical significance [26]. Characterized by a bitter taste and cooling properties, it exhibits meridian tropism toward the heart and kidney systems, demonstrating therapeutic effects including heat clearance from the heart, neurosedative action, essence consolidation, and hemostatic efficacy. These pharmacological attributes render it suitable for dual applications in both herbal medicine and functional tea formulations. Phytochemical analyses have identified isoquinoline alkaloids as its principal bioactive constituents, categorized into two structural classes: benzylisoquinoline alkaloids (e.g., neferine, liensinine, and isoliensinine) and aporphine alkaloids (e.g., nuciferine) [27,28]. These isoquinoline alkaloids exhibit notable pharmacological profiles, including demonstrated antidepressant efficacy [29], cardioprotective activity [30], and dual anti-inflammatory/antioxidant capabilities [29,31]. To further explore its potential, we developed a scalable, quality-controlled preparation of Nelumbinis Plumula total alkaloid extract (NPAE) using a Quality by Design approach. However, the content of its active alkaloids and its effects against IR-induced oxidative damage remain unclear.

This study combined phytochemical analysis with protective properties of NPAE in counteracting IR-induced cellular damage through oxidative stress regulation and inflammatory pathway inhibition. This integrated approach not only establishes a pharmaceutical-grade manufacturing framework but also deciphers the radioprotective mechanisms of NPAE at the molecular level, thereby bridging botanical research and clinical translation.

## 2. Materials and Methods

### 2.1. Chemicals and Reagents

Nelumbinis Plumula (lot no. 20220601) was purchased from TongRenTang Chinese Medicine (Beijing, China). The standards of Neferine (Nef, lot no. 10671), Liensinine (Lie, lot no. 14004), and Isoliensinine (IL, lot no. 9813) were purchased from Shanghai Standard Technology Co., Ltd. (Shanghai, China). Triethylamine was purchased from Beijing Innochem Technology Co., Ltd. (Beijing, China). Methanol and acetonitrile (HPLC grade solvents) were purchased from Thermo Fisher Scientific (Ward Hill, MA, USA). D001 resin was purchased from Beijing Solarbio Technology Co., Ltd. (Beijing, China). Bromocresol green (chemistry) and Potassium hydrogen phthalate were purchased from Adamas-beta Co., Ltd. (Shanghai, China). Dichloromethane and sodium hydroxide were purchased from Sinopharm Chemical Reagent Co., Ltd. (Beijing, China).

### 2.2. Preparation of Nelumbinis Plumula Total Alkaloid Extract (NPAE)

NPAE was prepared from Nelumbinis Plumula via ethanol reflux extraction and subsequent resin purification. The crude extraction was performed with 85% ethanol at 100 °C for 1.5 h per cycle, repeated three times with a liquid-solid ratio of 15:1 (mL/g). The resulting extract was then loaded onto a D001 resin column (Cat# M0047) after pH adjustment to 2.0. The purification conditions were a loading ratio of 10 mL resin per gram of extract, and elution with 2.0 bed volumes (BV) of 95% ethanol containing 1% NH_3_·H_2_O at a flow rate of 3 BV/h.

### 2.3. Analysis of Nef, Lie, IL and Total Alkaloid Content

The contents of three representative alkaloids (Nef, Lie and IL) were determined by high-performance liquid chromatography (HPLC) analysis. The standard of Nef, Lie, IL (1.0 mg/mL) or NPAE (5.0 mg/mL) was dissolved in 35% aqueous acetonitrile solution, and then were filtered prior to HPLC analysis. Chromatographic separation was performed using a Waters e2695 LC system (Waters Corp, Milford, MA, USA) with a NX-C18 110Å column (250 mm × 4.6 mm, 5 μm, Phenomenex, Torrance, CA, USA) for sample separation. Eluent A was 0.05% triethylamine aqueous solution, and eluent B was acetonitrile. The gradient program was as follows: 0–13 min, 35–60% B; 13–20 min, 60–80% B; 20–21 min, 80–95% B; 21–25 min, 95% B; 25–30 min 95–35% B. The column temperature was adjusted at 35 °C, flow rate was adjusted at 1.0 mL/min, and injection volume was 20 μL. The PDA detector was conducted at 282 nm and analytical curve with different concentrations of Nef, Lie and IL were plotted to calculate the content in NPAE (Table 1).

The content of total alkaloids was determined by spectrophotometric method that was conducted based on acid dye colorimetric method with minor modification [32]. Briefly, NPAE or Nef standard (10 mg) was weighed into 10 mL of volumetric flask and dissolved by methanol. A total of 0.4 mL of the sample solution was measured in a 50 mL dispensing funnel, and 7.0 mL of potassium hydrogen phthalate buffer (pH 4.5) was added and mixed well. Next, a 4.5 mL of bromocresol green solution was added and shook well, and subsequently an 8.0 mL of dichloromethane was mixed well and extracted. After standing for 2 h, the dichloromethane layer of the extracting solution was collected, and the absorbance was measured at 407 nm. The total alkaloid content was presented by the Nef standard curve (Table 1).

### 2.4. Cell Culture and γ-Irradiation

Human umbilical vein endothelial cells (HUVECs) were cultured in Dulbecco’s Modified Eagle Medium (DMEM; MeilunBio, Dalian, China, Cat# MA0213) supplemented with 5% (*v*/*v*) fetal bovine serum (FBS; Gibco, Grand Island, NY, USA, Cat# 16000-044) and 1% penicillin-streptomycin (100 U/mL; Macgene, Beijing, China, Cat# CC004). Cells were maintained at 37 °C in a humidified atmosphere containing 5% CO_2_. NPE and NPAE were dissolved in sterile dimethyl sulfoxide (DMSO; Innochem, Beijing, China, Cat# D3855) at stock concentrations of 100 mg/mL and 60 mg/mL, respectively. Stock solutions were aliquoted and stored at −20 °C, protected from light exposure. Working concentrations were prepared fresh prior to experiments, with final DMSO concentrations maintained below 0.2% (*v*/*v*) in all treatment groups. Cell irradiation was carried out at the ^60^Co γ-irradiation facility at the Beijing Institute of Radiation Medicine, Beijing, China. The irradiation was carried out at a dose rate of 78.5–86.6 cGy/min with samples placed 2 m from the ^60^Co to ensure uniform exposure.

### 2.5. Cell Viability Assay

Cell viability was analyzed using Cell Counting Kit-8 (CCK-8; Dalian Meilun Biotechnology Co., Ltd., Dalian, China, Cat# MA0218-3) according to the manufacturer’s instructions. Cells were treated with drugs in a 96-well plate. After treatment, the cells were incubated with CCK-8 working solution for 0.5 h at 37 °C in a humidified incubator containing 5% CO_2_, and the absorbance was then measured at 450 nm (Multiskan MK-3, Thermo Fisher Scientific, Ward Hill, MA, USA).

### 2.6. DNA Damage Assay

DNA damage was analyzed to γ-H2AX staining using a DNA Damage Detection Kit (Beyotime, Shanghai, China, Cat# C2035S) according to the manufacturer’s protocol, mounted with an anti-fade mounting medium, and subsequently examined γ-H2AX (Ex 490 nm/Em 520 nm) and DAPI (Ex 360 nm/Em 450 nm) using a fluorescence microscope (Olympus CKX53, Olympus Corporation, Tokyo, Japan).

Simultaneously, the content of 8-hydroxy-2′-deoxyguanosine (8-OHdG) in the cell culture supernatant was measured using a commercial enzyme-linked immunosorbent assay (ELISA) kit (Elabscience, Wuhan, China; Catalog No. E-EL-0028).

### 2.7. Intracellular ROS Level Assay

HUVECs were cultured in a 6-well plate, as mentioned above. After treatment for a specific time, DHE (Bioesn Biotechnologies Co., Ltd., Suzhou, China, Cat# BES-BK2782B) working solution (1:1000) and Hoechst 33,342 (ImmunoChemistry Technologies, LLC, Bloomington, MN, USA, Cat #639) working solution (1:200) were added to the cells incubating for 20 min under physiological conditions (37 °C, 5% CO_2_). Fluorescent images were acquired sequentially using the following filter sets: DHE (Ex 480 nm/Em 610 nm), Hoechst 33,342 (Ex 360 nm/Em 480 nm) under a fluorescence microscope.

### 2.8. Lactate Dehydrogenase (LDH) Release Assay

Cellular membrane integrity was quantitatively evaluated using a commercial Lactate Dehydrogenase (LDH) Cytotoxicity Detection Kit (Dojindo Molecular Technologies, Kumamoto, Japan, Cat# CK12). A total of 96 h after irradiation, 100 μL LDH working solution was added to each well and incubated for 15 min at 37 °C in dark. LDH activity was assessed by recording the absorbance at 490 nm and the LDH release rate was calculated as 100 × (experimental release − blank)/(release of IR − blank).

### 2.9. Transwell Permeability Assay

For the endothelial permeability assay, 5 × 103 HUVECs were plated on the upper chamber of 0.4 μm transwell inserts (Corning, NY, USA, Cat#353095). Complete culture medium was added to the lower chamber. HUVECs were grown for 2 days until they formed a confluent monolayer. A total of 48 h after irradiation, the upper chamber was supplemented with medium containing 0.5 mg/mL FITC-dextran for 1 h. Finally, BioTek Cytation 5 multi-mode microplate reader (BioTek Instruments, Inc., Winooski, VT, USA) was used to measure the amount of FITC-dextran that had diffused past the endothelial monolayer and into the bottom compartment at Ex/Em:490/520 nm.

### 2.10. Detection of Inflammatory Cytokine Level in Cell Supernatants

The levels of inflammatory mediators including IL-1β (MEIMIAN Biotechnology Co., Ltd., Jiangsu, China, Cat# MM-0181H1) and IL-18 (Dakewe Biotech Co., Ltd., Shenzhen, China, Cat# 1121802) in the cell supernatants were measured using ELISA. Following 96 h of post-radiation incubation, cell supernatants were collected through sequential centrifugation (1000× *g*, 20 min, 4 °C) and stored at −80 °C until analysis. In accordance with the protocols from the IL-1β and IL-18 kits, cytokine activity was assessed by recording the absorbance at 450 nm.

### 2.11. Flow Cytometry and Cell Pyroptosis Assay

Cellular pyroptosis was systematically evaluated using a FAM-FLICA™ Caspase-1 Assay Kit (ImmunoChemistry Technologies, LLC, Bloomington, MN, USA, Cat# 98) combined with propidium iodide (PI) membrane integrity staining. At 72 h post-irradiation, the cells were collected and washed with PBS and then stained with caspase-1 detection probe (FAM-YVAD-FMK). After 1 h of incubation, samples were washed with PBS and stained with PI solution for 10 min. Final analysis was performed using a BD FACSAria II flow cytometer (BD Biosciences, San Jose, CA, USA). Both FITC and PI were excited by the blue laser (488 nm), with FITC detected in the 530/30 nm channel and PI detected in the 695/40 nm channel. FLICA caspase-1 +/PI − were considered early pyroptosis, while FLICA caspase-1 +/PI − were considered late pyroptosis.

### 2.12. Western Blotting Analysis

At 72 h post-irradiation, cells were lysed using 200 μL RIPA buffer (Applygen Technologies Inc., Beijing, China; Cat# C1053-100) supplemented with protease inhibitor cocktail (1:100 *v*/*v*; Selleck Chemicals, Houston, TX, USA; Cat# B14001), which were lysed on ice for 30 min and then centrifuged at 12,000 rpm at 4 °C for 15 min.

Protein samples were resolved via sodium dodecyl sulfate-polyacrylamide gel electrophoresis (SDS-PAGE) under reducing conditions (120 V constant voltage, 90 min). After electrophoresis, the proteins on the gel were transferred to a PVDF membrane (0.45 μm pore size; Merck Millipore, Burlington, MA, USA, Cat# IPVH00010). Membranes were blocked with 5% (*w*/*v*) non-fat dried milk in Tris-buffered saline containing TBST for 1 h at 25 °C, followed by incubation with the following primary antibodies diluted in blocking buffer: occludin (Proteintech Group, Inc., Wuhan, China, Cat# 13409-1-AP), cleaved-caspase-1 (Abmart, Shanghai, China, Cat# P79884R2), pro-caspase-1 antibody (Abcam, Cambridge, UK, Cat# ab179515), GSDMD (Abcam, Cambridge, UK, Cat# ab219800) and GAPDH (Cell Signaling Technology, Danvers, MA, USA, Cat# 2118S). Primary antibody incubations were performed overnight at 4 °C with gentle agitation. Membranes were washed three times (10 min/wash) in TBST and subsequently incubated with horseradish peroxidase (HRP)-conjugated goat anti-rabbit IgG secondary antibody (ABclonal Biotechnology, Wuhan, China, Cat# AS014). Protein bands were visualized using Super ECL Detection Reagent kit (Yeasen Biotechnology, Shanghai, China, Cat# 36208ES76) according to manufacturer specifications. Chemiluminescent signals were captured with a ChemiDoc™ MP Imaging System (Bio-Rad Laboratories, Hercules, CA, USA). Band intensity quantification was performed using ImageJ software V1.8.0 [33].

### 2.13. Data Processing and Statistical Analysis

The results of the cell experiments and assays were collected from three independent replicates. All data are presented as the mean ± standard deviation (SD). Statistical significance (*p* value) was analyzed by one-way analysis of variance followed by Dunnett’s multiple comparison test using GraphPad Prism 10.1.2 (GraphPad Software Inc., San Diego, CA, USA). * *p* < 0.05; ** *p* < 0.01; *** *p* < 0.001; ns, non-significant.

## 3. Results and Discussion

### 3.1. Quantification of Nef, Lie, IL, and Total Alkaloid Content in NPAE

**Figure 1:**
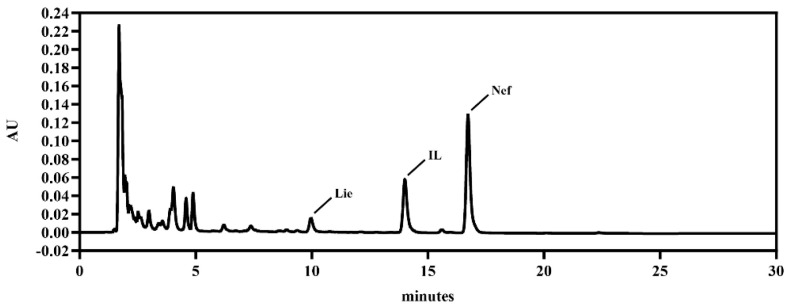
HPLC chromatogram of NPAE.

The contents of Nef, Lie, IL, and total alkaloids in NPAE were quantified using HPLC and spectrophotometric method, yielding values of 18.20 ± 0.25%, 1.40 ± 0.06%, 7.65 ± 0.15%, and 61.87 ± 1.32%, respectively (Figure 1).

Existing literature indicates that these alkaloids exhibit anti-inflammatory and antioxidant properties. For instance, Lie has been reported to suppress LPS-induced inflammatory response and attenuate apoptosis via modulation of the JNK/p38-ATF 2 axis [34]. IL mitigates osteoarthritis-induced joint damage by suppressing chondrocyte pyroptosis, which is achieved through regulation of the MAPK/NF-κB pathway, improvement of mitochondrial function, and reduction in intracellular oxidative stress [35]. Similarly, Nef decreases intracellular ROS levels and inhibits the ROS/NLRP3/Caspase-1 pathway, protecting against LPS-ATP-induced endothelial pyroptosis [36]. Given these documented mechanisms, NPAE with high contents of the active alkaloid ingredients may confer radioprotection through dual regulation of inflammatory and oxidative stress pathways. This supports further investigation into its potential as a therapeutic agent against radiation-induced injury.

### 3.2. Protective Effects of NPAE on H_2_O_2_-Induced Cytotoxicity and Intracellular Xidative Damage

**Figure 2:**
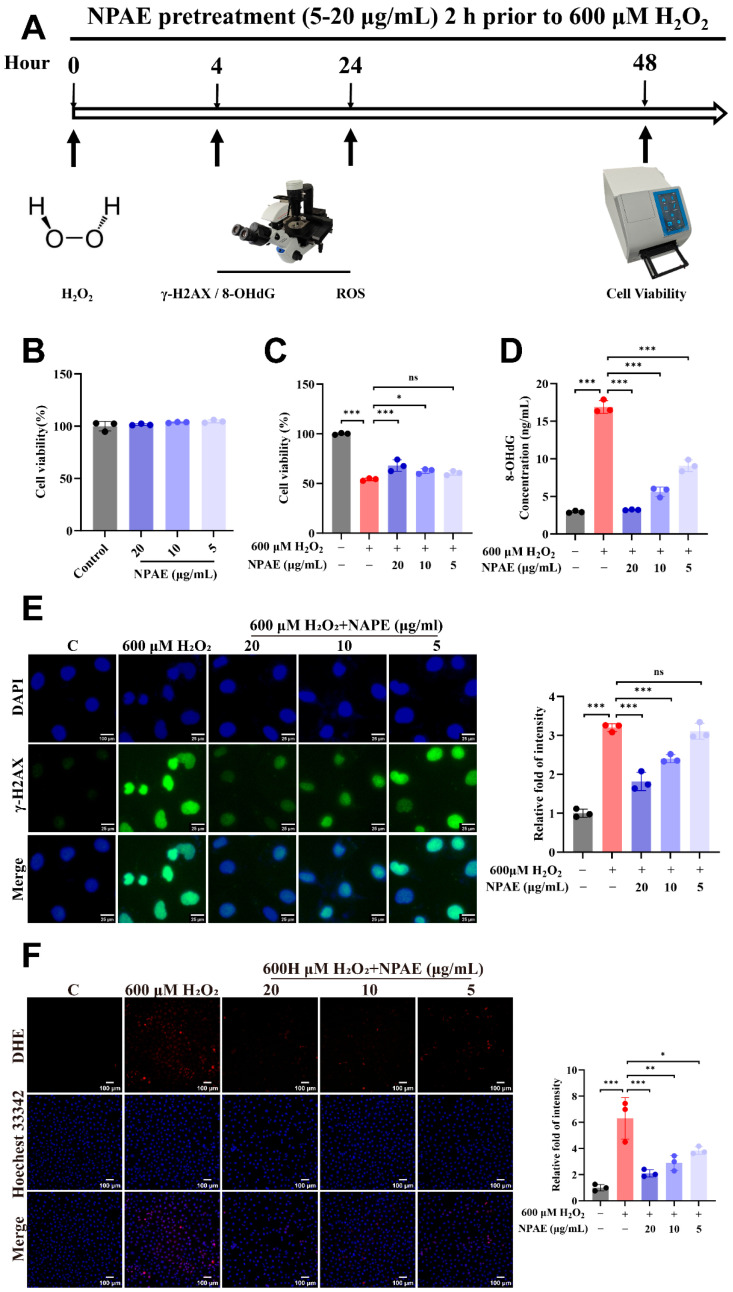
NPAE exerts antioxidative stress effects by enhancing cell viability, suppressing H_2_O_2_-induced ROS accumulation, and protecting against DNA damage. HUVECs received NPAE pretreatment (5–20 μg/mL) 2 h prior to 600 μM H_2_O_2_. Schematic representation of NPAE pretreatment experiments (**A**). Cell viability assay at 48 h post-treatment (**B**,**C**). ELISA analysis of 8-OHdG level at 4 h post-treatment (**D**). DAPI/γ-H2AX co-staining visualized DNA damage with fluorescence intensity analysis at 4 h post-treatment (**E**). DHE/Hoechst 33,342 co-staining visualized ROS accumulation with fluorescence intensity analysis at 24 h post-treatment (**F**). * *p* < 0.05, ** *p* < 0.01, *** *p* < 0.001 vs. IR group; ns indicated non-significant.

To simulate the indirect damage predominant in radiation injury, which is primarily mediated by ROS [9,37], an oxidative stress model was established in HUVECs using H_2_O_2_. Based on this model, the protective efficacy of NPAE pretreatment against H_2_O_2_-induced oxidative stress damage was assessed (Figure 2A).

Non-cytotoxic working concentrations of NPAE were first determined via CCK-8 cytotoxicity screening (Figure 2B). Subsequent analysis revealed that NPAE (20 μg/mL) significantly attenuated H_2_O_2_-induced cytotoxicity in a concentration-dependent manner. At 48 h post-H_2_O_2_, NPAE restored HUVECs viability to 68.01 ± 5.81% (*p* < 0.001), compared to 54.01 ± 1.29% in the model group (Figure 2C).

We further evaluated oxidative DNA damage using 8-OHdG, a well-established biomarker for oxidative stress and disease risk. H_2_O_2_ exposure induced substantial intracellular ROS generation (Figure 2F), resulting in significant DNA damage, as evidenced by guanine base oxidation (8-OHdG formation, 16.90 ± 0.85 ng/mL, *p* < 0.001; Figure 2D) and histone H2AX phosphorylation (increased γ-H2AX expression; Figure 2E). Notably, NPAE pretreatment effectively reduced ROS levels and dose-dependently suppressed the expression of both 8-OHdG (3.21 ± 0.05 ng/mL, *p* < 0.001) and γ-H2AX.

The use of an H_2_O_2_-induced oxidative stress model offers distinct advantages for dissecting the mechanisms of indirect radiation damage. By exogenous introducing H_2_O_2_, this approach recapitulates the key post-irradiation ROS burst while circumventing the complexities associated with direct radiation effects on biomolecules [38]. This strategy ensures high controllability, precise dosing, and well-defined timing, thereby providing a robust platform for systematically elucidating the regulatory properties of natural radioprotective agents such as NPAE.

### 3.3. Protective Effects of NPAE on IR-Induced Cytotoxicity and Intracellular Oxidative Damage

**Figure 3:**
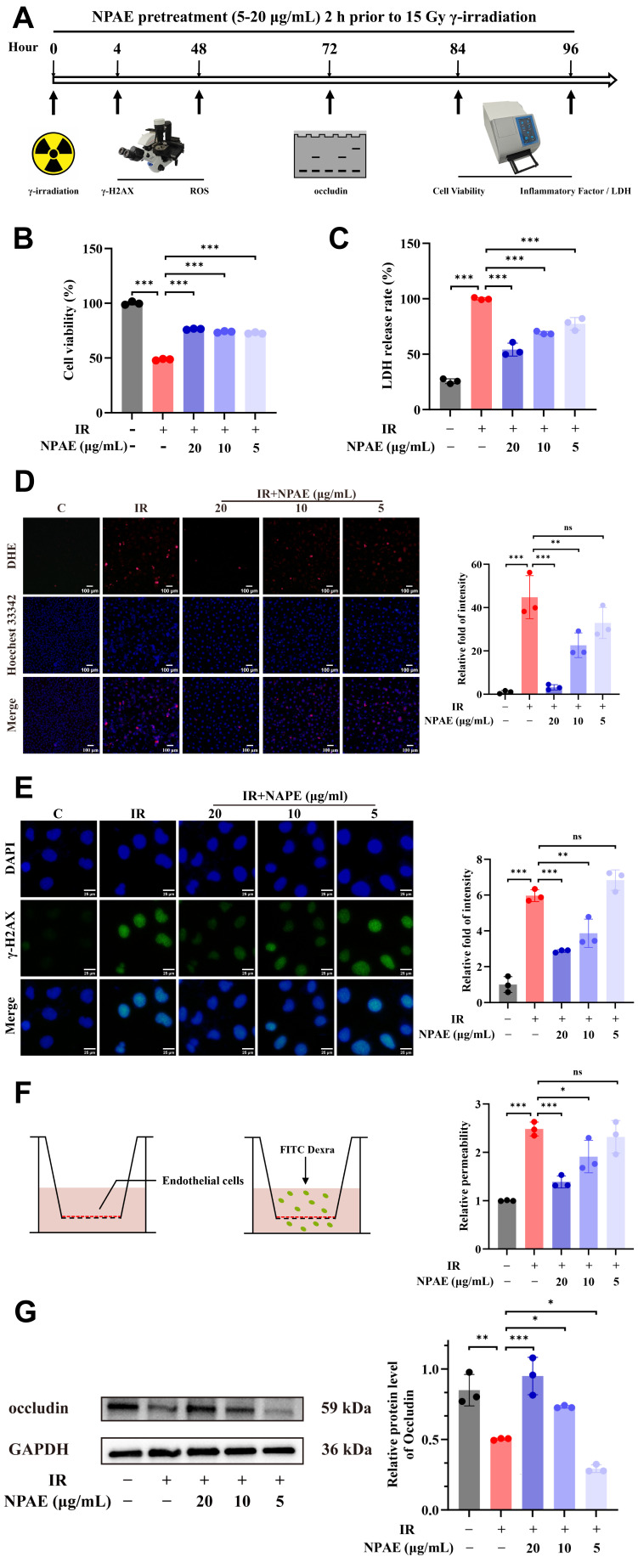
NPAE exerts dual radioprotective effects by enhancing cellular viability and mitigating irradiation-induced ROS accumulation. HUVECs received NPAE pretreatment (5–20 μg/mL) 2 h prior to 15 Gy irradiation. Schematic representation of NPAE pretreatment experiments (**A**). Cell viability assay at 84 h post-irradiation (**B**). Quantitative LDH release analysis in supernatants 96 h post-irradiation (**C**). DHE/Hoechst 33,342 co-staining visualized ROS accumulation with fluorescence intensity analysis at 48 h post-irradiation (**D**). DAPI/γ-H2AX co-staining visualized DNA damage with fluorescence intensity analysis at 4 h post-irradiation (**E**). Transwell permeability assay for quantitative assessment of monolayer integrity at 48 h post-irradiation (**F**). Western blot detection of occludin processing quantification at 72 h post-irradiation (**G**). * *p* < 0.05, ** *p* < 0.01, *** *p* < 0.001 vs. IR group; ns indicated non-significant; GAPDH served as loading control for immunoblotting.

To evaluate the radioprotective capacity of NPAE, an in vitro model of radiation injury was established by exposing HUVECs to 15 Gy irradiation. Using this model, we investigated the cytoprotective effects of NPAE pretreatment against irradiation-induced cellular damage (Figure 3A).

Cell survival analysis indicated that NPAE (20 μg/mL) significantly attenuated IR-induced cytotoxicity in a concentration-dependent manner. At 84 h post-irradiation, NPAE restored HUVEC viability to 76.38 ± 0.52% (*p* < 0.001), compared to 48.59 ± 0.73% in the radiation-only group (Figure 3B). These results quantitatively demonstrate the efficacy of NPAE in enhancing endothelial cell survival under radiation stress.

Consistent with improved cell viability, LDH release in cell culture supernatants was measured at 96 h post-irradiation. A significant increase (*p* < 0.001) in extracellular LDH was observed in the irradiated group, whereas NPAE pretreatment (5–20 μg/mL) dose-dependently suppressed this elevation, with a 45.84 ± 5.93% reduction at 20 μg/mL compared to the IR group (Figure 3C).

To explore the antioxidant mechanism of NPAE, intracellular ROS levels were assessed using DHE fluorescence. Quantitative image analysis at 48 h post-irradiation showed a pronounced increase in ROS-specific fluorescence in irradiated cells relative to non-irradiated control group (*p* < 0.001). Pretreatment with 20 μg/mL NPAE significantly attenuated radiation-induced ROS accumulation (*p* < 0.001), with a clear dose–response relationship (Figure 3D).

We further evaluated DNA damage and endothelial monolayer integrity. Immunofluorescence staining revealed that irradiation significantly increased γ-H2AX foci formation, which was effectively suppressed by NPAE in a dose-dependent manner (Figure 3E). FITC-dextran permeability assays showed that radiation severely disrupted monolayer integrity, leading to increased permeability. NPAE pretreatment mitigated this effect by upregulating the tight junction protein occludin (Figure 3F,G). Collectively, these data indicate that NPAE confers multi-faceted protection against radiation-induced endothelial cell injury.

### 3.4. Protective Effects of NPAE on IR-Induced Cellular Pyroptosis

**Figure 4:**
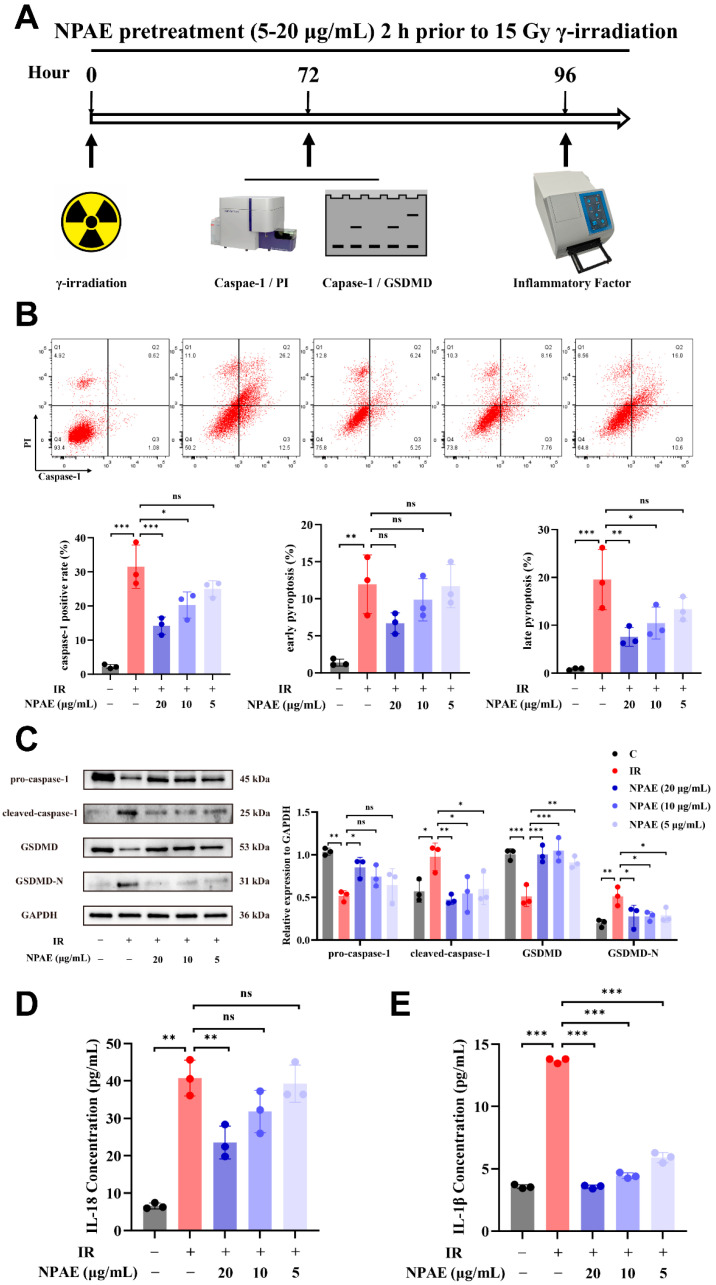
NPAE protects endothelial cells against IR-induced damage by suppressing the pyroptosis pathway through downregulation of key executioner proteins. Schematic representation of NPAE pretreatment experiments (**A**). Caspase-1/PI dual staining quantifying early (Q2: caspase-1 +/PI −) and late (Q3: caspase-1 +/PI +) pyroptotic populations at 72 h post-irradiation (**B**). Western blot detection of pro-caspase-1, its activated fragment cleaved caspase-1 and GSDMD proteolytic processing quantification at 72 h post-irradiation (**C**). ELISA analysis of IL-18 secretion (**D**) and IL-1β secretion (**E**) 96 h post-irradiation. * *p* < 0.05, ** *p* < 0.01, *** *p* < 0.001 vs. IR group; ns indicated non-significant; GAPDH served as loading control for immunoblotting.

We next evaluated whether NPAE pretreatment could modulate radiation-induced pyroptosis (Figure 4A). Given that radiation-triggered pyroptosis is character by plasma membrane rupture and the release of damage-associated molecular patterns (DAMPs), such as LDH, IL-1β, and mitochondrial DNA, thereby propagating inflammatory cascades [39]. We assessed this form of programmed necrosis using caspase-1/PI dual staining flow cytometry protocol at 72 h post-irradiation (Figure 4B). Relative to unirradiated controls, irradiated HUVECs exhibited a 14-fold increase (*p* < 0.001) in active caspase-1 + cells, accompanied by elevated pyroptotic indices (early stage: 11.95 ± 3.95%; late stage: 19.57 ± 6.33%). NPAE pretreatment (20 μg/mL) significantly suppressed caspase-1 activation by 17.30 ± 6.84% (*p* < 0.001) and reduced late-stage pyroptosis to 7.59 ± 1.98% (*p* < 0.01), with concentration-dependent effects also observed at 10 μg/mL (9.11 ± 7.16% reduction, *p* < 0.05).

To further elucidate the molecular mechanisms of NPAE-mediated protection, we examined key executors of the pyroptotic pathway. Immunoblotting analysis showed that IR (15 Gy) induced proteolytic cleavage of both GSDMD and caspase-1 compared with unirradiated cells (Figure 4C). NPAE (20 μg/mL) pretreatment effectively restored GSDMD integrity and suppressed caspase-1 activation in a concentration-dependent manner.

In addition, multiplex ELISA analysis of secreted DAMPs at 96 h post-irradiation revealed significant radiation-induced increases in IL-1β (13.69 ± 0.20 pg/mL vs. control 3.57 ± 0.15 pg/mL, *p* < 0.001) and IL-18 (40.77 ± 4.78 pg/mL vs. control 6.56 ± 0.78 pg/mL, *p* < 0.01). NPAE pretreatment dose-dependently reduced the levels of these cytokines (20 μg/mL: IL-1β 3.56 ± 0.14 pg/mL, 74% reduction, *p* < 0.001 vs. IR group; IL-18 23.54 ± 4.39 pg/mL, 42% suppression, *p* < 0.01 vs. IR group; Figure 4D,E).

To establish a standardized in vitro radiobiological model, we implemented 15 Gy irradiation in HUVECs, a well-characterized system for studying radiation-induced cellular injury and vascular endotheliopathy [40]. Through systematic time-course characterization (24–96 h post-irradiation), we identified phase-specific cellular responses: (1) no significant morphological alterations or viability changes in latent phase (0–24 h post-irradiation), yet damage to DNA had already occurred; (2) cytoplasmic edema with increase in cell volume and membrane blebbing observed via microscopy in transition phase (48 h post-irradiation); (3) plasma membrane rupture and cytoplasmic debris accumulation in microscopic fields in execution phase (72 h post-irradiation); (4) significant viability reduction detected by CCK-8 at 84 h post-irradiation. The observed temporal dissociation—molecular activation peaking at 48–72 h, while viability loss became evident only at 84 h—highlights the importance of endpoint-specific experimental designs when evaluating radioprotective agents. Our findings demonstrate that systematic time-course analysis can reveal distinct kinetic profiles between early molecular events and later functional outcomes. This approach provides a clear framework for comprehensively elucidating the mechanisms of action of radioprotective agents.

Oxidative stress, as a principal mediator of radiation-induced bystander effects, represents a cornerstone in the pharmacological evaluation of radioprotective agents [38]. Our results demonstrate that NPAE significantly alleviates IR-generated ROS accumulation. This finding is evidenced by a marked reduction in DHE fluorescence intensity (*p* < 0.001). Thus, the data confirms NPAE’s ability to mitigate radiation-associated oxidative injury. ROS act as pivotal second messengers that orchestrate redox-sensitive signaling cascades, including those regulating programmed cell death. Growing evidence points to intricate crosstalk between ROS dynamics and regulated necrosis pathways [37,41]. Mechanistically, radiation-induced mitochondrial ROS overproduction triggers endothelial pyroptosis through dual pathways involving immunogenic activation and direct membrane permeabilization [42]. In this study, the DHE probe was employed to assess the overall changes in cellular oxidative stress levels. Although the signal may be subject to minor interference from other reactive oxygen species, this detection method is well-established, reliable, and has been widely adopted and recognized in the fields of radiation biology and oxidative stress research [43,44,45]. Furthermore, considering the high sensitivity of mitochondria to oxidative stress and the predominant origin of intracellular ROS from mitochondrial dysfunction, the observed increase in DHE fluorescence, while indirect evidence, strongly suggests the accumulation of mitochondria-derived superoxide anion. Direct evidence, however, is still lacking. Future studies will employ mitochondria-targeted ROS probes to verify and clarify the critical role of mitochondrial ROS in this process.

Furthermore, ROS overproduction serves as an initiating signal for the release of damage-associated molecular patterns (DAMPs) [46]. This redox perturbation promotes inflammasome assembly, proteolytic maturation (including caspase-1 activation and GSDMD-N forming), and amplification of cytokine release (e.g., IL-1β and IL-18) [39]. Our mechanistic investigation further revealed that NPAE exerts potent anti-pyroptotic effects. Immunoblot analysis showed that NPAE (20 μg/mL) suppressed radiation-induced proteolytic activation of caspase-1 and GSDMD cleavage. This pyroptotic paradigm was quantitatively confirmed by caspase-1/PI co-staining flow cytometry (Q2) following irradiation (19.57 ± 6.33%), an effect that was substantially suppressed by NPAE pretreatment (7.59 ± 1.98%, *p* < 0.01). These findings establish the multimodal radioprotective mechanism of NPAE, spanning the restoration of redox homeostasis and the suppression of inflammatory activation. We found that the peak of radiation-induced ROS burst (48 h) preceded the significant activation of caspase-1 (72 h), thereby supporting the role of ROS as an upstream trigger. Furthermore, radiation induced proteolytic cleavage of caspase-1 and processing of GSDMD, which are hallmark events of pyroptotic execution [47]. Although these observations strongly suggest activation of the NLRP3 inflammasome, this study did not directly assess the activation status of NLRP3 or ASC. Future work will directly examine the effect of NPAE on the NLRP3 inflammasome and employ NLRP3 knockdown or specific inhibitors to evaluate their impact on caspase-1 activation and cell death.

The cellular response to radiation is complex, with pathways such as apoptosis, necroptosis, and pyroptosis potentially coexisting and interacting [48,49]. We focused on elucidating the protective effect and mechanism of NPAE against radiation-induced endothelial pyroptosis. Although other death pathway-specific markers were not systematically examined, the coherent evidence presented here—comprising caspase-1 cleavage, cleavage of its substrate GSDMD, and the release of downstream pro-inflammatory cytokines (IL-1β/IL-18)—strongly confirms the marked activation of the pyroptosis pathway in this model. Future studies will include more comprehensive detection of markers for different death pathways to systematically compare their relative contributions to radiation-induced endothelial injury.

The radioprotective effects and molecular mechanisms elucidated in this study are based on NPAE—a multi-component system. IR-induced damage to organisms is a typical multi-target, multi-pathway, and multi-stage complex systemic process, involving DNA damage, oxidative stress burst, inflammatory cascade reactions, and activation of multiple programmed cell death pathways, among others [50,51,52]. As a natural multi-component system containing various alkaloids (such as neferine, liensinine, isoliensinine) and other potential active constituents, the advantage of NPAE lies in its ability to act synergistically through different components on various nodes of the damage network, thereby potentially providing more comprehensive protection [53,54]. Furthermore, although monomeric compounds such as neferine and liensinine have been confirmed to possess distinct bioactivity [34,36], their total chemical synthesis routes are extremely lengthy, involve complex steps, and result in low overall yields, leading to high production costs that hinder their suitability for large-scale drug development. In contrast, preparing a standardized total alkaloid extract (NPAE) using mature extraction and purification processes offers significant advantages in technical feasibility and economic cost. In summary, the endothelial and vascular barrier-protective effects demonstrated by NPAE suggest its potential value as a vascular protective agent in scenarios involving occupational or accidental radiation exposure.

## 4. Conclusions

**Figure 5:**
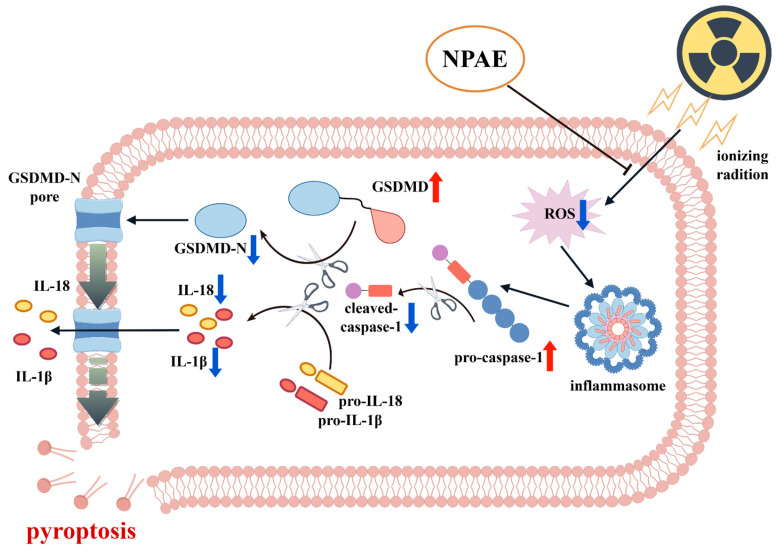
The potential mechanisms of NPAE in mitigating IR-induced cell pyroptosis via targeting the ROS/caspase-1/GSDMD axis (By figdraw.com).

Utilizing a high-purity alkaloid-enriched extract (NPAE) from Nelumbinis Plumula, which was standardized to a total alkaloid content of 61.87 ± 1.32%, this study revealed its novel role in mitigating radiation-induced endothelial injury. We demonstrated that NPAE pretreatment (20 μg/mL) significantly counteracted H_2_O_2_-induced oxidative stress and attenuated IR-induced pyroptotic cell death, restoring cell viability to 76.38 ± 0.52% (*p* < 0.001 vs. IR group). The radioprotective mechanism involves the restoration of redox homeostasis and suppression of the inflammasome activation cascade. Collectively, these results establish NPAE as a phytotherapeutic agent targeting the ROS/caspase-1/GSDMD axis (Figure 5) and reveal a potential new treatment strategy for irradiation-associated complications.

## Figures and Tables

**Table 1:** Linearity and standard curve of three representative alkaloids and total alkaloids.

Index	Regression Equation	*r*	Range of Linearity (μg/mL)
Nef	y = 2 × 10^7^x − 17,583	1.0000	3.75–240.00
Lie	y = 2 × 10^7^x − 38,602	0.9999	3.75–240.00
IL	y = 2 × 10^7^x − 40,407	0.9999	3.75–240.00
Total alkaloids	y = 2.8881x − 0.0693	0.9992	76.00–534.00

## Data Availability

The original contributions presented in this study are included in the article. Further inquiries can be directed to the corresponding authors.
